# Supplementary figures and images for: Quantifying cryptic Symbiodinium diversity within Orbicella faveolata and Orbicella franksi at the Flower Garden Banks, Gulf of Mexico
Source: PeerJ. 2014 May 13;2:e386. doi: 10.7717/peerj.386 (PMC4034615; doi:10.7717/peerj.386)

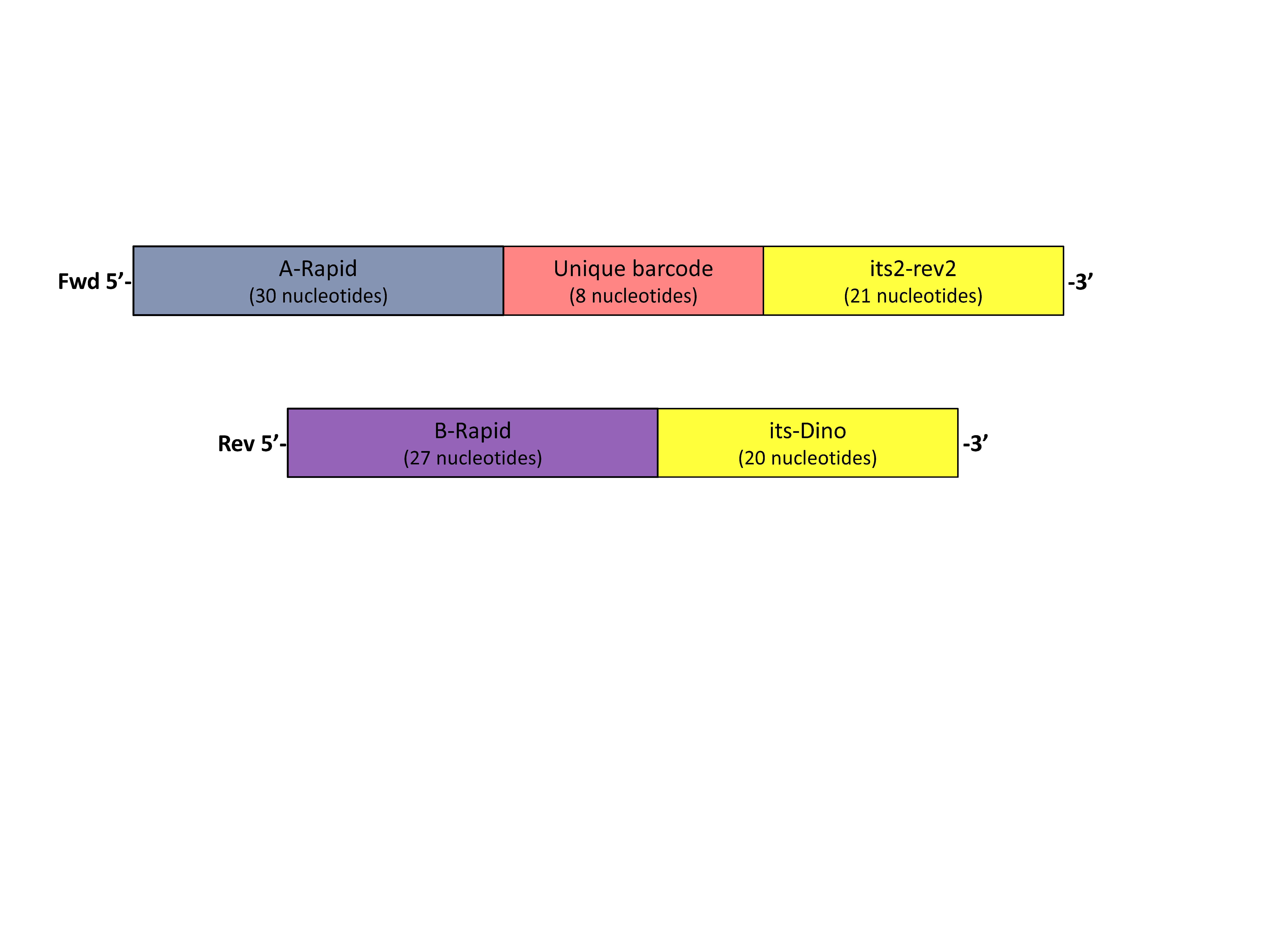

Supplement: Figure S1 [file peerj-02-386-s001.png]

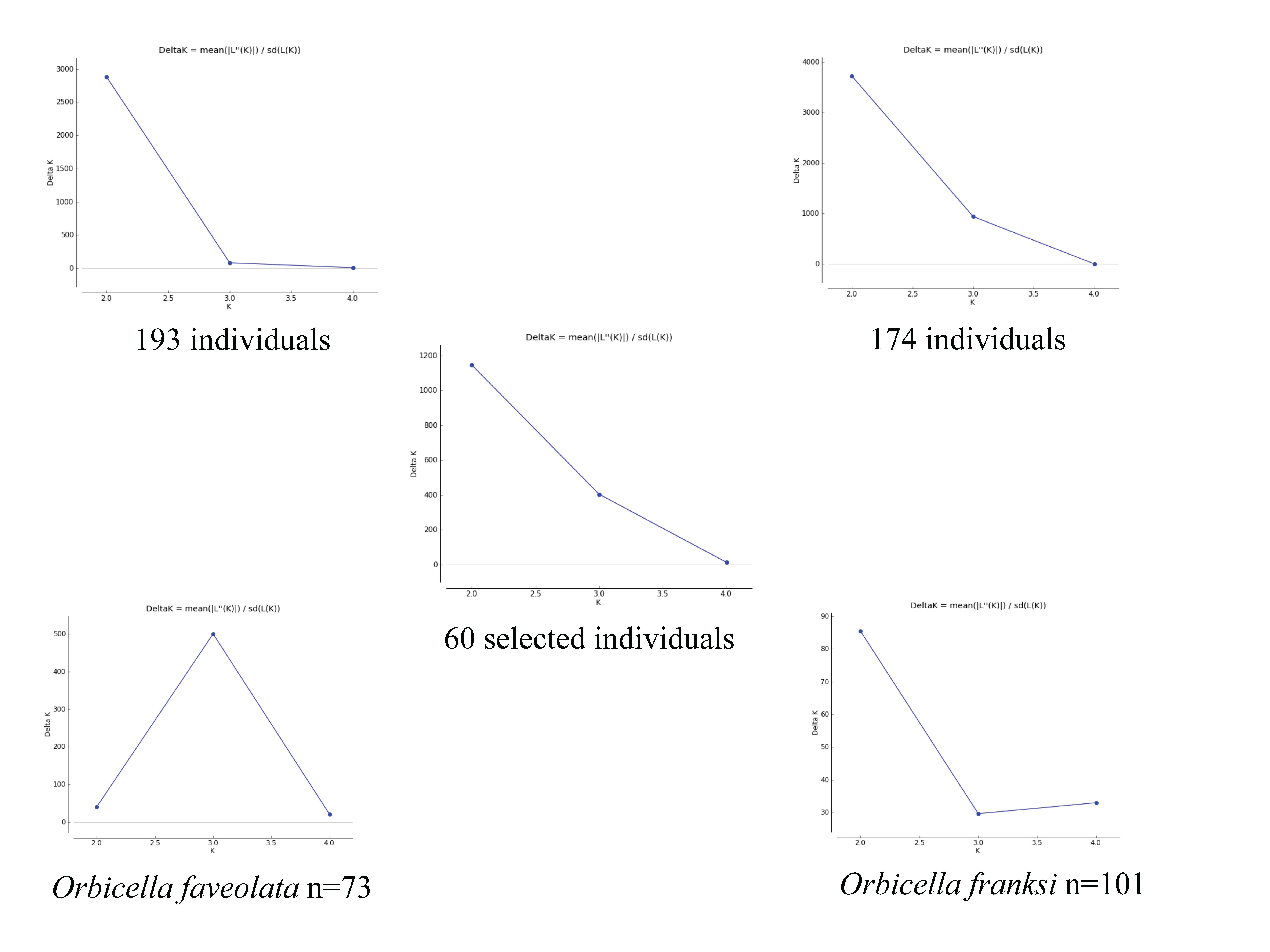

Supplement: Figure S2 [file peerj-02-386-s005.png]

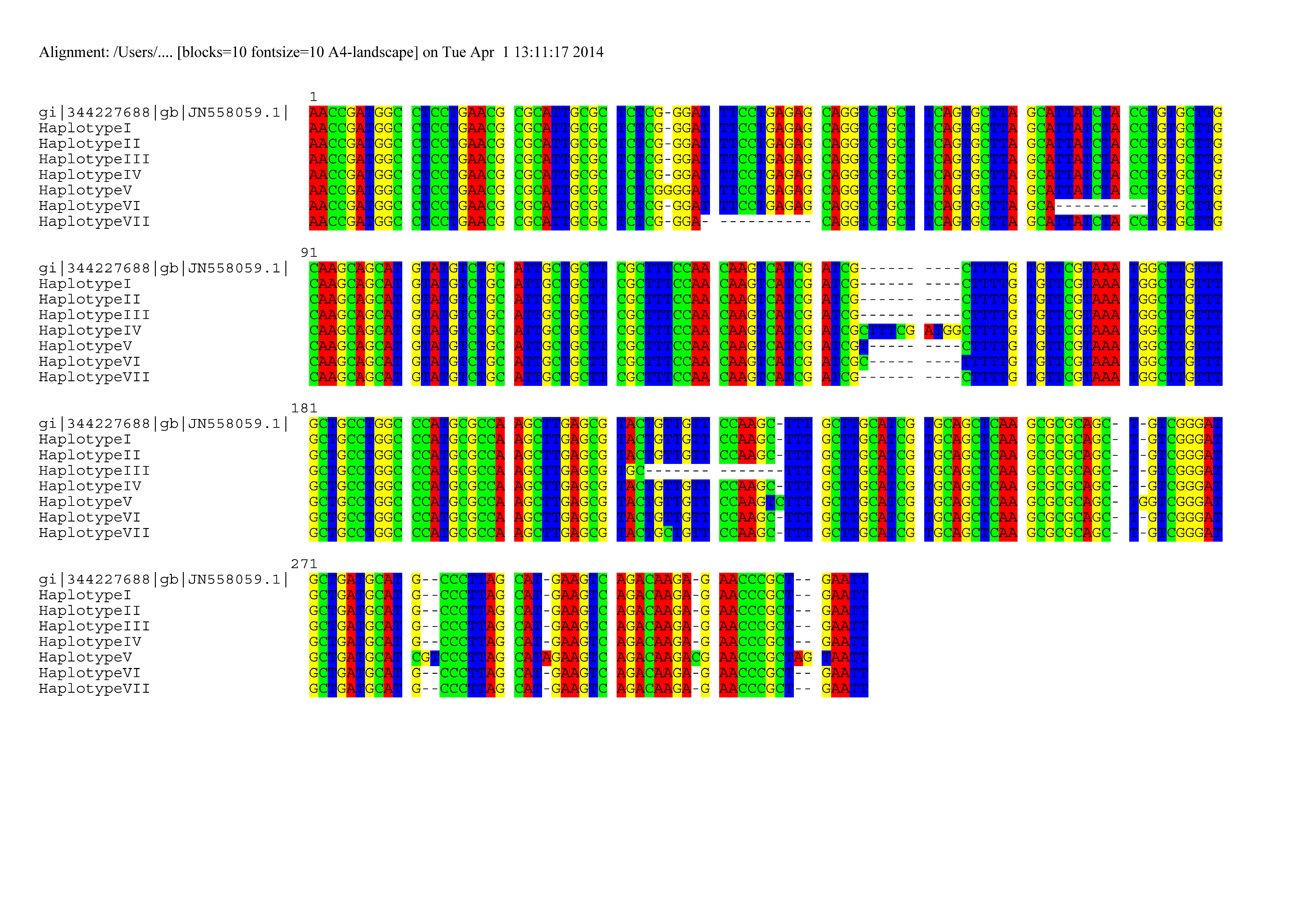

Supplement: Figure S3 [file peerj-02-386-s007.png]

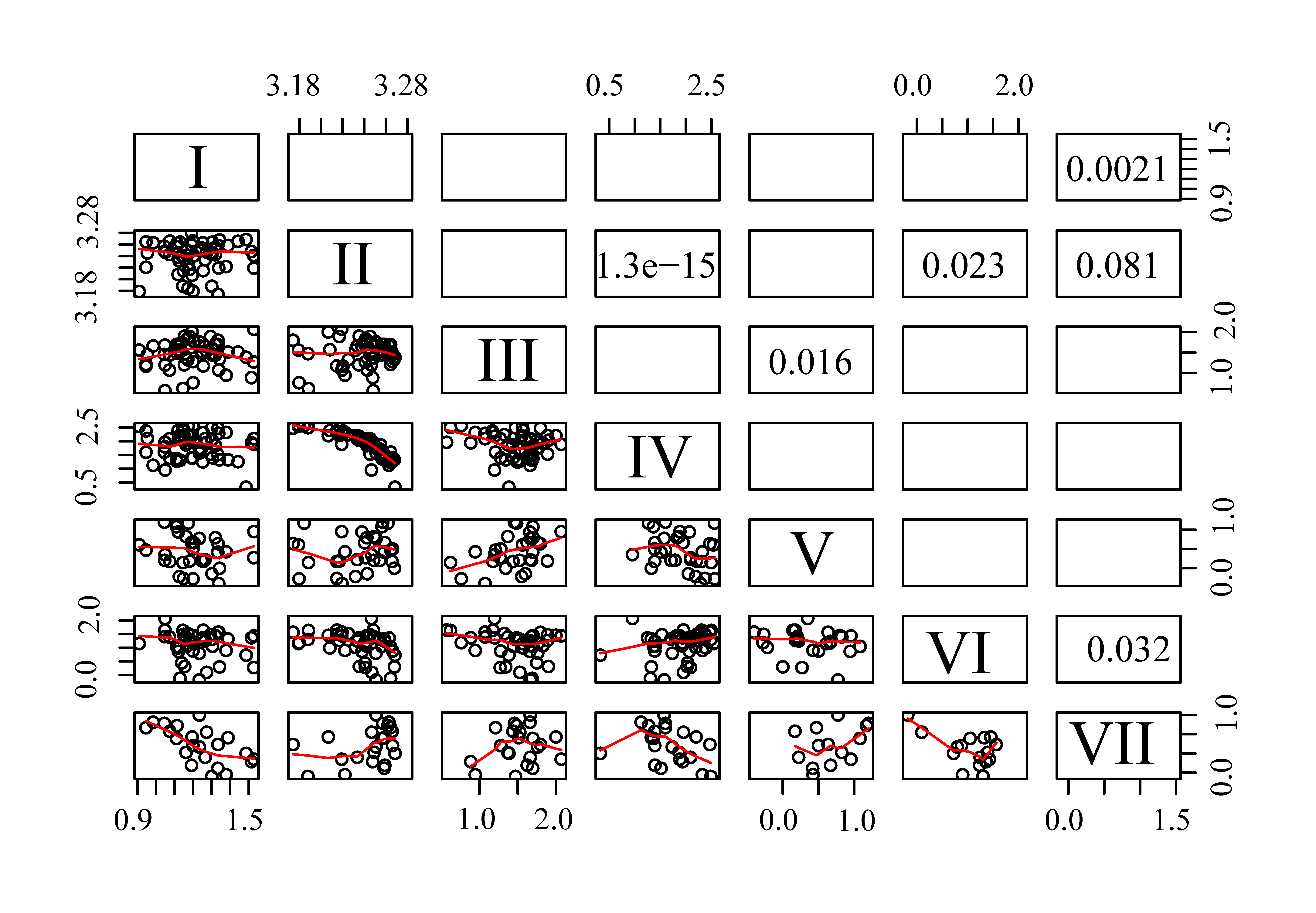

Supplement: Figure S4 — Scatter plots with lowess-smoothing lines are shown in lower triangle and p-values < 0.1 are shown in the upper triangle. [file peerj-02-386-s009.png]
