# Supplementary material for: Quantifying cryptic Symbiodinium diversity within Orbicella faveolata and Orbicella franksi at the Flower Garden Banks, Gulf of Mexico
Source: PeerJ. 2014 May 13;2:e386. doi: 10.7717/peerj.386 (PMC4034615; doi:10.7717/peerj.386)
Supplement: Table S1 [file peerj-02-386-s006.docx]

|  |  | Bank:Species | Bank:Species |
| --- | --- | --- | --- |
| Haplotype IV |  | east:*O. faveolata* | east:*O. franksi* |
|  | east:*O. faveolata* | NA | 0.084788945 |
|  | east:*O. franksi* | 0.89804683 | NA |
|  | west:*O. faveolata* | 0.01567222 | 0.005357879 |
|  | west:*O. franksi* | 0.07730115 | 0.020078324 |
|  |  | west:*O. faveolata* | west:*O. franksi* |
|  | east:*O. faveolata* | -0.5438136 | -0.4269689 |
|  | east:*O. franksi* | -0.6286026 | -0.5117579 |
|  | west:*O. faveolata* | NA | 0.1168447 |
|  | west:*O. franksi* | 0.8120322 | NA |
| Haplotype VI |  | east:*O. faveolata* | east:*O. franksi* |
|  | east:*O. faveolata* | NA | -0.32404926 |
|  | east:*O. franksi* | 0.485795916 | NA |
|  | west:*O. faveolata* | 0.002146626 | 0.01567222 |
|  | west:*O. franksi* | 0.062058148 | 0.48579592 |
|  |  | west:*O. faveolata* | west:*O. franksi* |
|  | east:*O. faveolata* | -1.238575 | -0.6543721 |
|  | east:*O. franksi* | -0.9145257 | -0.3303229 |
|  | west:*O. faveolata* | NA | 0.5842029 |
|  | west:*O. franksi* | 0.128632 | NA |
| Haplotype VII |  | east:*O. faveolata* | east:*O. franksi* |
|  | east:*O. faveolata* | NA | -0.664176119 |
|  | east:*O. franksi* | 0.52600063 | NA |
|  | west:*O. faveolata* | 0.02111635 | 0.005357879 |
|  | west:*O. franksi* | 0.02111635 | 0.005357879 |
|  |  | west:*O. faveolata* | west:*O. franksi* |
|  | east:*O. faveolata* | 1.2667628 | 1.2389624 |
|  | east:*O. franksi* | 1.9309389 | 1.9031386 |
|  | west:*O. faveolata* | NA | -0.0278004 |
|  | west:*O. franksi* | 0.9808469 | NA |
