# Supplementary material for: Quantifying cryptic Symbiodinium diversity within Orbicella faveolata and Orbicella franksi at the Flower Garden Banks, Gulf of Mexico
Source: PeerJ. 2014 May 13;2:e386. doi: 10.7717/peerj.386 (PMC4034615; doi:10.7717/peerj.386)
Supplement: Table S2 — Percent transfer is the percent of each helix from the model B1 ITS-2 structure that was found within the secondary structure for a novel ITS-2 sequence. [file peerj-02-386-s008.docx]

| Target Number | Template Number | Structure Number | Transfer % Helix 1 | Transfer % Helix 2 | Transfer % Helix 3 | Transfer % Helix 4 | Transfer % Helix Ø |
| --- | --- | --- | --- | --- | --- | --- | --- |
| Haplotype I | 1 | 1 | 100 | 100 | 100 | 100 | 100 |
| Haplotype II | 1 | 1 | 100 | 100 | 100 | 100 | 100 |
| Haplotype III | 15 | 1 | 100 | 100 | 72.73 | 100 | 93.18 |
| Haplotype IV | 1 | 1 | 100 | 100 | 100 | 100 | 100 |
| Haplotype V | 14 | 1 | 100 | 100 | 85.71 | 100 | 96.43 |
| Haplotype VI | 1 | 1 | 100 | 100 | 100 | 100 | 100 |
| Haplotype VII | 1 | 1 | 100 | 100 | 100 | 100 | 100 |
| B1 JN558059.1 | 1 | 1 | 100 | 100 | 100 | 100 | 100 |
